# Non-Coding RNAs in Cancer: Structure, Function, and Clinical Application

**DOI:** 10.3390/cancers17040579

**Published:** 2025-02-08

**Authors:** Éva Márton, Alexandra Varga, Dóra Domoszlai, Gergely Buglyó, Anita Balázs, András Penyige, István Balogh, Bálint Nagy, Melinda Szilágyi

**Affiliations:** 1 Department of Human Genetics, Faculty of Medicine, University of Debrecen, H-4032 Debrecen, Hungary; marton.eva@med.unideb.hu (É.M.); varga.alexandra@med.unideb.hu (A.V.); domoszlai.dora@med.unideb.hu (D.D.); buglyo.gergely@med.unideb.hu (G.B.); penyige@med.unideb.hu (A.P.); balogh@med.unideb.hu (I.B.); nagy.balint@med.unideb.hu (B.N.); 2 Department of Integrative Health Sciences, Institute of Health Sciences, Faculty of Health Sciences, University of Debrecen, H-4032 Debrecen, Hungary; balazs.anita@etk.unideb.hu; 3 Division of Clinical Genetics, Department of Laboratory Medicine, Faculty of Medicine, University of Debrecen, H-4032 Debrecen, Hungary

**Keywords:** cancer, cancer diagnostics, cancer therapy, RNA, RNA detection, miRNA, lncRNA, circRNA, snRNA, snoRNA

## Abstract

We are on the brink of a paradigm shift in both theoretical and clinical oncology. Genomic and transcriptomic profiling, alongside personalized approaches that account for individual patient variability, are increasingly shaping discourse. Discussions on the future of personalized cancer medicine are mainly dominated by the potential of non-coding RNAs (ncRNAs), which play a prominent role in cancer progression and metastasis formation by regulating the expression of oncogenic or tumor suppressor proteins at transcriptional and post-transcriptional levels; furthermore, their cell-free counterparts might be involved in intercellular communication. Non-coding RNAs are considered to be promising biomarker candidates for early diagnosis of cancer as well as potential therapeutic agents. This review aims to provide clarity amidst the vast body of literature by focusing on diverse species of ncRNAs, exploring the structure, origin, function, and potential clinical applications of miRNAs, siRNAs, lncRNAs, circRNAs, snRNAs, snoRNAs, eRNAs, paRNAs, YRNAs, vtRNAs, and piRNAs. We discuss molecular methods used for their detection or functional studies both in vitro and in vivo. We also address the challenges that must be overcome to enter a new era of cancer diagnosis and therapy that will reshape the future of oncology.

## Simple Summary

The field of non-coding RNAs has growing interest in cancer research since these molecules play a prominent role in the regulation of cancer progression. Furthermore, they are considered to be promising biomarker candidates and therapeutic targets that might revolutionize cancer diagnostics and therapy in the future. Here, we aimed to provide an overview of the functions and possible clinical applications of non-coding RNAs in cancer, including miRNAs, siRNAs, lncRNAs, circRNAs, snRNAs, snoRNAs, eRNAs, paRNAs, YRNAs, vtRNAs, and piRNAs. We also present molecular methods for their detection and functional characterization.

## 1. Introduction

Sequencing the human genome led to the unexpected recognition that only 1–2% of the human genome is responsible for coding proteins [1]. The remainder of the genome was categorized as “junk”. However, 74% of the human genome is transcribed into RNA molecules, raising the question of whether these “junk RNAs” have any function in cells. Nowadays, they are called non-coding RNAs (ncRNA) as they are not translated to proteins, and it is generally accepted that they play relevant roles in the regulation of cellular physiology [2]. They are traditionally classified by their size. Some species are longer than 200 nt (e.g., long non-coding RNAs [lncRNAs]; circular RNAs [circRNAs]), while small non-coding RNAs (sncRNAs) are 18–200 nt long (e.g., microRNAs [miRNA], PIWI-interacting RNA [piRNA], small nucleolar RNAs [snoRNAs], small interfering RNAs [siRNAs], small nuclear RNAs [snRNAs], YRNAs, and vaultRNA [vtRNA]) [3,4]. By function, they may be classified as housekeeping ncRNAs, which are highly expressed in every cell type and perform essential functions in various cellular processes. Apart from well-known RNA species such as rRNAs, tRNAs, snRNAs, and snoRNAs, notable ncRNAs have regulatory functions and are involved in the initiation of transcription (promoter-associated RNAs [paRNAs], enhancer RNAs [eRNAs]), RNA degradation (endogenous siRNAs), the maintenance of genome integrity by silencing transposons (piRNAs), or in the regulation of gene expression (miRNAs, lncRNAs, circRNAs) [5,6,7,8,9,10,11]. In this review, we discuss the exact contribution of these ncRNAs to cancer progression, and we also present strategies for their possible clinical application. In addition, we present molecular methods that can be applied for their detection and functional characterization in vitro and in vivo.

## 2. The Role of RNA Networks in Cancer

Regulation of gene expression is mediated via the cooperation of several ncRNAs, with miRNAs considered the central players. The binding of miRNAs to mRNA targets results in the degradation of the mRNA or a block of translation; thus, miRNAs are considered negative regulators [6]. Fine-tuning of gene expression is mediated by RNA interactions: (i) one miRNA targets several mRNAs; (ii) one mRNA might be targeted by several miRNAs; (iii) lncRNAs and circRNAs might serve as sponges for miRNAs that decrease the availability of miRNAs in the cells [12]. Their interaction is well explained by the competing endogenous hypothesis [12,13]. A miRNA response element (MRE) is a target sequence recognized by a miRNA on a specific mRNA. MicroRNAs typically have a number of target mRNAs that possess MREs. According to the competing endogenous hypothesis, mRNAs possessing MREs of the same miRNA compete with each other for the available miRNAs and influence each other’s translation. This represents a crosstalk through the language of nucleic acids in cells [13]. Pseudogenes might also compete for miRNAs with the mRNA transcripts of their ancestral genes. Furthermore, other ncRNAs (e.g., lncRNAs and circRNAs) possess MREs as well and compete with each other and with mRNAs for miRNAs, resulting in an extensive regulatory crosstalk in the transcriptome [12,13]. These functional networks are necessary for the normal physiology of cells. Perturbations might lead to diseases of the cardiovascular and immune systems, neurological disorders, or cancer [14,15,16].

**Figure 1:**
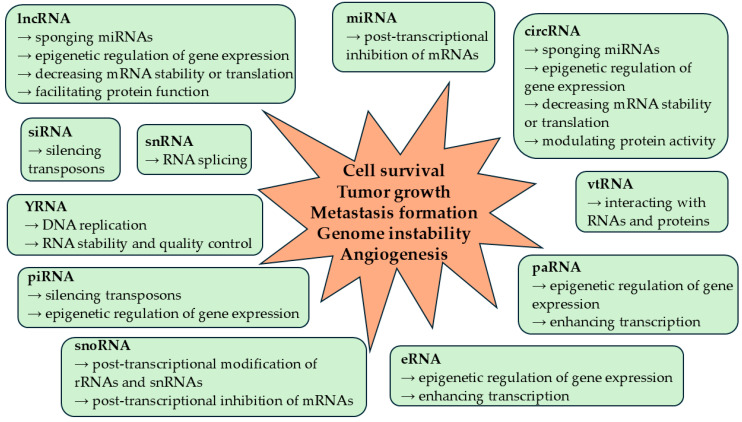
The role of various ncRNAs in the development of cancer. Non-coding RNAs influence the expression of proteins involved in pathways related to tumor progression at both transcriptional and post-transcriptional levels.

Non-coding RNAs are key players in the development of cancer as they affect the production of oncogenic and tumor suppressor proteins (Figure 1). MicroRNAs suppressing the production of tumor suppressor proteins are known as oncomiRs, while those that inhibit the synthesis of oncogenic proteins are tumor suppressor miRNAs [17]. Long non-coding RNAs and circRNAs might also have diverse functions in cancer development. They may promote cancer progression by enhancing the transcription of oncogenes through DNA interactions, affecting mRNA translation and the stability of oncogenic transcripts, sponging tumor suppressor miRNAs, or modulating the activity of proteins by serving as scaffolds or decoys [16]. PIWI-interacting RNA-PIWI complexes might transcriptionally silence tumor suppressor proteins via aberrant DNA methylation or histone modification [18]. Furthermore, the inappropriate expression of eRNAs, paRNAs, snoRNAs, and snRNAs was also associated with cancer development via the dysregulation of gene expression or RNA modifications (Figure 1) [3,4,10,19].

## 3. Cell-Free Non-Coding RNAs

Non-coding RNAs function not only intracellularly but also extracellularly. Non-codingRNAs may leave cells and appear in the extracellular matrix or in body fluids (as cell-free non-coding RNAs [cf-ncRNAs]) (i) via active transport (by vesicles including exosomes and microvesicles), (ii) in association with RNA-binding proteins (HDL, LDL, AGO2), or (iii) as a result of cell death (cell necrosis/apoptosis) [20,21,22,23].

RNAs released actively and packed into exosomes and microvesicles are thought to play pivotal roles in cell-to-cell communication. Cell-free miRNAs may enter surrounding cells via endocytosis, membrane fusion, or specific cell-surface receptors and influence their gene expression [21,22,24]. Exosomal miRNAs may be responsible for the transition of normal tissue microenvironments into pro-tumorigenic microenvironments [25], an effect most well-studied in neuroblastoma by co-culture experiments. These showed a transfer of exosomal miR-21 from neuroblastoma cells to monocytes, upregulating miR-155 via TLR8 and NF-κB. Monocyte-derived miR-155 is then transferred back to neuroblastoma cells, where it targets TERF1, impacting telomerase activity and length, which is linked to drug resistance and poor prognosis [26]. Furthermore, cf-miRNAs might promote the transition of fibroblasts that support cancer progression by reordering the extracellular matrix and secreting various molecules (e.g., cytokines, growth factors, chemokines) [21]. Such cancer-associated fibroblasts may release exosomes (to be taken up by cancer cells) carrying ncRNA cargo such as miR-21 [27]. Cell-free miRNAs may also modulate the immune response by supporting the polarization of tumor-associated macrophages as well as angiogenesis by modulating endothelial cells [21]. Long non-coding RNAs also contribute to cell-to-cell communication. Based on the available evidence, lncRNAs are packaged in exosomes and interact with both neighboring and distant cells through active transport processes. Once inside, they may affect normal cell functions, inducing tumorigenesis, promoting angiogenesis, and stimulating tumor growth and drug resistance [23,28].

Extracellular argonaute 2 (AGO2) has been proposed as a key component of extracellular RNA-induced silencing complexes suppressing mRNAs in recipient cells, but there is still no consensus on whether they are mainly transported inside or outside of extracellular vesicles [29]. HDLs transport host-derived small RNAs, such as miRNAs, tRNAs, rRNAs, and snRNAs, and nonhost small RNAs originating from the microbiome, a process often disturbed in disease, resulting in proinflammatory pathways getting triggered when the lipoproteins are engulfed by macrophages [30].

Apoptotic bodies and other vesicles from dying cells encapsulate a wide range of content, including small molecules, DNA fragments, micronuclei, and even entire organelles such as mitochondria. They have also been reported to carry various cfRNA species such as rRNAs, U1 RNAs, and YRNAs, some of which may have altered nucleotide compositions and secondary structures, including a high frequency of U-rich motifs and unstable folding elements [31]. These immunostimulatory RNAs are believed to function in tandem with self-antigen fragments to regulate processes such as autoimmunity and even cancer [32].

## 4. Isolation and Detection of Non-Coding RNA Molecules

The detection of ncRNAs is more challenging than detecting mRNAs due to the following reasons: (i) short ncRNAs are discarded along with RNA fragments in conventional RNA extraction procedures, (ii) the short size of ncRNAs makes cDNA synthesis and detection challenging, and (iii) the detection of cf-ncRNAs is further hindered by their low concentration in body fluids. Despite these difficulties, several methods have been developed for the reliable detection of ncRNAs [33,34,35]. Choosing an RNA extraction method retaining short (<200 bp) RNA sequences is essential. Such RNA extraction kits, as well as reagents for enhancing RNA stability, are commercially available. An easy-to-use example is the solid phase spin-column method involving RNase-free silica membranes that are provided by several vendors. Conventional RNA detection methods such as Northern blot, RT-qPCR, microarray analysis, and next-generation sequencing (NGS) can be used for ncRNA detection. Since Northern blotting is a time-consuming, multi-step procedure [36], RT-qPCR is the generally preferred method. The bias caused by the short sequences of most ncRNAs can be overcome by either (i) the RNA being reverse transcribed into cDNA using miRNA-specific stem and loop primers before amplification; (ii) miRNAs being elongated with a poly-A tail during reverse transcription with a common primer, followed by amplification using universal and miRNA-specific primer sets [37,38]. Normalization of miRNA expression levels against a reference is necessary. Selecting proper references requires thorough consideration and is often a limiting factor in published studies. Using housekeeping ncRNAs (e.g., RNU6) as references is a common choice, but their expression might also change in cancer and might show variability among patients. Taking such changes into account, the optimization of references is a crucial step during study design, and a combination of references may yield better results than any single reference RNA on its own [35]. Primer assays for qPCR-based detection of lncRNAs and circRNAs are also available [39,40]. For the latter, the method relies upon circRNA’s higher resistance to degradation by RNases. Purification involves RNAse R treatment to remove linear RNA molecules. Any remaining linear poly(A)^+^ RNAs are removed by oligo(dT) beads. Following reverse transcription, circRNAs can be amplified by PCR using circRNA-specific divergent primers [41]. However, RNAse R might digest high molecular weight circRNAs while leaving linear RNAs with stable secondary structures intact. This procedure is also not feasible for the detection of cf-circRNAs due to their low concentration levels. Other alternative methods are also available, including RT-ddPCR, ligation-based PCR assays, rolling cycle amplification, and stem-loop primer-based systems for eliminating artifacts and increasing specificity [40]. Droplet digital PCR (ddPCR) is a good choice for the detection of cf-ncRNAs expected to be found in low concentrations in the samples. This method is based on partitioning nucleic acids into water-in-oil emulsion microdroplets that might contain 0, 1, or more template molecules, according to the Poisson distribution. These droplets also contain reagents and are used as amplification chambers. Fluorescence-emitting droplets are quantified, resulting in thousands of data points that enhance the power of statistical analysis [42].

For global transcriptomic profiling, microarray and NGS methods are available. Limitations of microarray-based methods include relatively low sensitivity and specificity, the need for high RNA input, and unsuitability for identifying novel ncRNAs. Distinguishing miRNAs with high levels of sequence similarity is also problematic [43]. Bead-array-based flow cytometric RNA profiling is more cost-effective than the solid-surface array. The method involves probes ligated to uniquely colored polystyrene beads to which the biotinylated miRNAs are hybridized. This is followed by streptavidin–phycoerythrin staining and analysis with flow cytometry. Quantification is carried out by determining bead color and intensity [44]. NGS methods are steadily gaining ground in the expression profiling of sncRNA species. This method includes cDNA library preparation followed by sequencing. The short sequence of ncRNAs represents the greatest challenge in sequencing, which may be addressed by extending these molecules via ligation or polyadenylation to provide binding sites for primers used in reverse transcription and amplification. However, this step also introduces additional bias as adaptors might cause artificial changes in ncRNA content due to their differing affinities for ncRNAs, consequently affecting PCR amplification efficiency. The method might detect particular miRNAs at varying efficiencies, leading to misleading results in extreme cases. This is especially challenging in cf-ncRNAs detection due to their low concentrations. To mitigate such effects, two-adaptor ligation (including ligation of the polyadenylated 3′ end and 5′ end adaptor) and polyadenylation-based ligation-free approaches may be considered [45]. The method TGIRT-seq applies a structure-tolerant reverse transcriptase, allowing sequencing of full-length ncRNAs by avoiding ligation bias caused by structural differences [46]. Single-cell RNA sequencing methods have also been optimized for small RNAs, enabling the monitoring of sncRNAs in individual cells rather than capturing an average of large cell populations, as seen in conventional bulk sequencing. Such methods of monitoring ncRNA expression are especially useful in the context of tumor tissues displaying heterogeneity or studies of cells with prominent functions in cancer progression (e.g., cancer stem cells). Parallel single-cell small RNA sequencing (PSCSR-seq) provides an optimized method via increased ligation efficiency, improved ligation adapters, and a nanowell chip [47]. The analysis of RNA sequencing data also introduces some bias. One significant challenge is the alignment of short sequencing reads with a high degree of stringency to a reference genome. Furthermore, RNA sequencing pipelines utilize general databases (such as GENCODE or RefSeq) to assign sequencing reads to specific genes and are not suitable for aligning reads that map to non-annotated genes. The use of specialized databases (such as RNAcentral or sRNA tools) integrating annotation from multiple sources may facilitate more accurate ncRNA annotations [48,49]. Another problem is the biased distribution of reads, with the majority of reads aligning to a limited number of loci within the genome and the possibility of a single read mapping to multiple locations. The presence of isoforms, particularly in the case of miRNAs, introduces another level of complexity. To account for such biases, a combination of methods (e.g., NGS methods and RT-qPCR) is strongly recommended for ncRNA detection in order to reduce the occurrence of misleading results.

## 5. Functional Studies of Non-Coding RNAs

### 5.1. In Vitro Methods for Studying Non-Coding RNA Function

**Figure 2:**
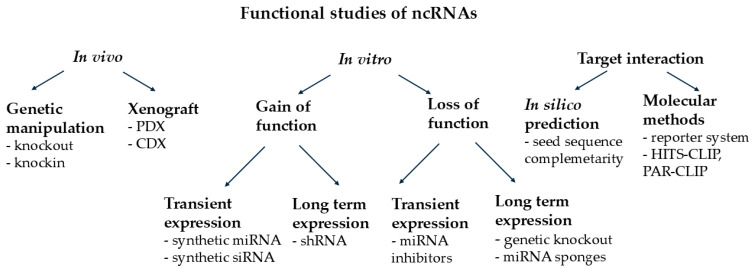
Methods for functional characterization of ncRNAs.

Due to recent advancements in bioinformatics and molecular biology, understanding the exact role of ncRNAs in the development of diseases is getting within reach. As a first step, it requires in vitro studies (Figure 2). Most reports focusing on miRNA function use miRNA mimics (synthetic, double-stranded oligonucleotides containing a guide and a passenger strand and capable of incorporating into the RISC complex) as a simple method for studying the transient expression of miRNAs in cells. Chemical modifications of miRNA mimics ensure the interaction of the guide strand with the Ago proteins [50,51]. MicroRNA mimics are available from several manufacturers and are delivered by a simple transfection method. Small interfering RNAs may also be applied to in vitro gene silencing. They specifically target a given gene product, binding with full complementarity, resulting in ~80% gene silencing potential via a single cleavage of the mRNA. In contrast, miRNA mimics require only partial complementarity, which may lead to the degradation of the target or a block of translation with ~30–60% gene suppression potential [52,53]. For this reason, siRNAs are the preferred choice when more comprehensive and precise gene silencing is needed, whether for research purposes or therapeutic applications. MicroRNAs may act synergistically due to their network-forming ability, leading to a cumulative impact where individually mild effects add up through the presence of multiple miRNAs regulating the same targets or several targets involved in the same biological process [52,54]. The drawback is that high cytoplasmic concentrations of miRNA mimics or siRNAs might have off-target effects that may only be overcome through careful design and optimization of the transfection procedure. Furthermore, they may become diluted in the cell culture as a result of cell divisions, making multiple rounds of transfections necessary, hence limiting their application in long-term experiments [52,54].

Using short hairpin RNAs (shRNAs) enables more efficient long-term expression of siRNAs and miRNAs and is well-suited for creating stable clones. The main forms of shRNAs are stem-loop-structured or miRNA-adapted ncRNAs encoded by a DNA vector. Simple stem-loop shRNAs are akin to endogenous pre-miRNAs and are similarly cleaved by the Dicer enzyme after their transcription. MicroRNA-adapted shRNAs resemble pri-miRNAs, processed by both Drosha and Dicer enzymes [55,56]. MicroRNA-adapted shRNAs contain miRNA-like mismatches and are less toxic to cells due to their more efficient processing. The DNA vector can be introduced into target cells by transfection methods (e.g., electroporation, lipid transfection) or viral transduction (e.g., adenovirus, lentivirus). Lentiviral shRNA vectors (e.g., SMARTvector shRNA) can be effectively used in almost any cell type, including non-dividing cells and cells that are difficult to transfect for other reasons [57]. Viral transduction also promotes reliable integration into the genome, allowing stable expression even in an inducible knockdown system. Selection markers enable the identification of successfully transfected or transduced cells, while fluorescent markers are used to screen cells expressing shRNAs (e.g., the GFP-green fluorescent protein). However, a high level of shRNA expression might saturate the endogenous RNAi system or promote off-target effects. These unintended events can be minimized by the careful design of the DNA vector (increasing specificity or using inducible promoters) [56,57,58]. Another popular method is the use of nanoparticle-conjugated shRNAs (nanoconjugates), which enhance stability and resistance against nucleases. Examples of such systems include lipid-, gold-, and silica-based nanoparticles and theranostic nanocarriers [55].

The possible effects of miRNA loss can be studied through genetic knockouts, such as those generated using the CRISPR/Cas9 system [50]. The application of miRNA inhibitors specifically binding endogenous miRNAs is another simple way of studying these effects. Such inhibitors include antisense oligonucleotides (ASOs) forming stable heteroduplexes with miRNAs and preventing their interaction with target mRNAs. Locked nucleic acids (LNAs) are efficient due to their stability and binding affinity for their targets [50,59]. MicroRNA sponges are also available and well-suited for the creation of stable loss-of-function clones. These are encoded by DNA plasmids, which might include promoters for constitutive or tissue-specific expression, as well as fluorescent reporters that allow for quantitative analysis. Sponges typically contain 10 binding sites and might inhibit a whole family of miRNAs sharing a common seed sequence [60].

### 5.2. In Vivo Methods for Studying Non-Coding RNA Function

Although in vitro methods are useful for the functional characterization of ncRNAs, understanding their actual contribution to cancer development requires in vivo studies (Figure 2). A number of animal models have been used for this purpose, including *C. elegans*, *D. melanogaster*, *D. rerio,* and *M. musculus*. For cancer research, the latter (as a mammalian model) is generally preferred [61]. Traditional approaches include knockouts for miRNA-coding loci, constructs for overexpressing miRNAs, and editing miRNA sequences. Transgenic mouse models can be generated via microinjection, homologous recombination, Cre-LoxP, Flp-FRT, and inducible systems, or CRISPR/Cas technologies. Microinjection involves introducing a transgene into a fertilized egg to produce an offspring that expresses the transgene. In homologous recombination, embryonic stem cells are employed to replace an endogenous gene with a transgene. The Cre-LoxP and Flp-FRT systems are site-specific recombination mechanisms useful for controlling gene expression, while inducible systems like Tet-ON/OFF and Cre-ER(T) allow reversible control of transgene expression. The CRISPR/Cas system enables precise targeting of specific miRNA sequences, minimizing unintended effects [61,62]. We need to consider that such animal experiments represent technical challenges and involve financial investment; thus, the 3R principle (reduce, refine, replace) may prove invaluable during study design. Additionally, reproducibility might also be a limiting factor due to biological variability among animal populations [63].

A simpler method for studying the role of ncRNAs in tumor development and/or invasion in vivo is the use of xenograft mouse models [61]. These involve injecting human tumor cells into immunodeficient mice, Nude (Foxn1, Nu/Nu), SCID, NOD/SCID, or NOD-scid IL2Rcnull (NSG) for later analysis [64]. The injection may be administered subcutaneously (SC), intraperitoneally (IP), or to a more specific site, e.g., intrabursally (IB) in ovarian cancer studies (into the bursa of the mouse ovary). SC models are suitable for imaging but not for studying metastasis, while IP and IB models perform better in that respect, even allowing ascite formation [64,65]. However, the technical execution of such procedures is challenging due to the risks associated with anesthesia, surgical complications, and infections, which are particularly hazardous for nude mice due to their immunosuppressed state. Furthermore, breeding such mice requires more care (e.g., sterile conditions and aseptic techniques, with minimum handling to avoid infections) compared to conventional animal models. There are two main types of xenograft models: (i) cell-line-derived xenograft (CDX), where human tumor cell lines are implanted into the mouse, and (ii) patient-derived xenograft (PDX), where the tumor tissue is taken directly from a patient and implanted into the mouse. The PDX model is suitable for studying the effect of specific mutations or the patterns of ncRNA expression in a patient and may also help assess treatment options [66]. However, CDX models have many advantages in the context of ncRNA studies. Cell lines offer a consistent and reproducible source of tumor cells, enabling comparison across different research settings. Furthermore, they can be readily implanted into mice, allowing for faster study initiation and lower costs compared to PDX models. The greatest advantage is the potential for in vitro genetic manipulation before implantation (e.g., transfection with miRNA mimics, siRNAs, shRNAs, or ASOs), allowing specific in vivo characterization of ncRNAs. This allows researchers to observe tumor growth, metastasis, and response to treatments in a more complex environment than a cell culture. Additionally, the model may be used to test the efficacy of anti-cancer drugs. Limitations include an inability to fully replicate the human immune response against cancer and a lack of representation of human tumors’ full complexity. Furthermore, PDX models might also be hampered by the limited availability of patient tumor samples, by the variability of tumor engraftment success, and by tumor heterogeneity [67,68,69].

### 5.3. Studying MicroRNA–Messenger RNA Interactions

MicroRNA–mRNA interactions can be studied using bioinformatics tools and in vitro methods (Figure 2). Several databases are available that provide reliable information about ncRNA sequences, expression in several cancer types, and their interaction with mRNAs or with other ncRNAs (Table 1). MicroRNA target predictions made by bioinformatic platforms are mainly based on sequence similarities between the 3′ UTR region of mRNAs and the seed sequence of miRNAs, incorporating thermodynamic analyses of miRNA–mRNA complexes to assess hybridization stability. The number of predicted interactions can be reduced by ignoring false positives estimated by the accessibility of the binding sites in both mRNAs and miRNAs, or by taking evolutionary conservation into account, as advantageous interactions are often conserved. Recently developed machine-learning methods may also help identify biologically relevant interactions [70]. Although computer predictions help to interpret results obtained from high-throughput detection methods and promote study design, they often identify an overwhelming amount of non-relevant target interactions. As a consequence, interactions considered to be important need to be confirmed by a reporter system in which the entire 3′ UTR region of the target gene is cloned downstream of a reporter gene (e.g., GFP or luciferase) in a plasmid that allows the study of all predicted miRNAs. Cells are transfected with this construct accompanied by miRNA mimics. Interactions with miRNAs lead to decreased expression of the reporter gene, resulting in lower fluorescence intensity. MicroRNA-controlled suppression of protein levels can be further validated by Western blot, ELISA, or immunocytochemical assays [70,71].

High-throughput methods are also available for the detection and faster validation of miRNA–mRNA interactions. These are based on the immunoprecipitation of Ago proteins with highly selective antibodies, followed by the isolation and sequencing of the RNA incorporated into the RISC complex. This results in two datasets (miRNA-Ago and mRNA binding site-Ago) that can be further explored using bioinformatic methods [70]. The high-throughput sequencing of RNA isolated by crosslinking immunoprecipitation (HITS-CLIP) method involves 254 nm UV radiation applied to covalently cross-link the RNA–protein complexes at their interacting site before immunoprecipitation and often results in more reproducible results [72]. An alternative method is the photoactivable-ribonucleoside-enhanced CLIP (PAR-CLIP), in which photoactivatable ribonucleosides such as 4-thiouridine (4SU) or 6-thioguanine (6SG) are randomly incorporated into RNA during transcription and are excited by 365 nm UV light that increases crosslinking efficiency. It also leads to a characteristic mutation at the site of crosslinking (G-to-A for 6SG and T-to-C for 4SU) during reverse transcription, which allows more accurate identification of interaction sites [73].

**Table 1:** Examples of databases for ncRNA studies in cancer. “Disease association” refers to databases providing ncRNA expression patterns in diseases, including cancer. “Expression” refers to databases containing expression data of ncRNAs in various samples/sources.” Gene, genome, annotation” refers to databases providing sequence or genomic location data. “Interaction” refers to databases providing information on interactions between RNAs.

Database Name	Full Name	Data Type	Database Category	Source	Found Year	Reference
HMDD	Human MicroRNA Disease Database	miRNA	Disease association	https://www.cuilab.cn/hmdd accessed on 5 November 2024	2008	[74]
TANRIC	The atlas of noncoding RNAs in cancer	lncRNA	Disease association	http://bioinformatics.mdanderson.org/main/TANRIC:Overview accessed on 5 November 2024	2015	[75]
dbDEMC	Database of Differentially Expressed MiRNAs in human Cancers	miRNA	Disease association	https://www.biosino.org/dbDEMC/index accessed on 5 November 2024	2010	[76]
miRCancer	microRNA Cancer Association Database	miRNA	Disease association	http://mircancer.ecu.edu/ accessed on 5 November 2024	2013	[77]
LncACTdb	Database of lncRNA-associated competing triplets	miRNA, lncRNA	Disease association	http://bio-bigdata.hrbmu.edu.cn/LncACTdb/ accessed on 5 November 2024	2015	[78]
lncRNAfunc	A knowledgebase of lncRNA function in human cancer	lncRNA	Disease association	https://ccsm.uth.edu/lncRNAfunc/ accessed on 5 November 2024	2021	[79]
lnCaNet	lncRNA–cancer gene co-expression networks	lncRNA	Disease association	https://lncanet.bioinfo-minzhao.org/web/lncRNACancer_v1/index.html accessed on 5 November 2024	2016	[80]
NoncoRNA	non-coding RNAs	piRNA, miRNA, lncRNA, circRNA	Disease association	http://www.ncdtcdb.cn:8080/NoncoRNA/ accessed on 5 November 2024	2020	[81]
CIRCpedia	A database of circular RNAs	circRNA	Expression	http://www.picb.ac.cn/rnomics/circpedia accessed on 5 November 2024	2016	[82]
exoRBase	mRNAs, lncRNAs, and circRNAs in extracellular vesicles	lncRNA, circRNA	Expression	http://www.exorbase.org/ accessed on 5 November 2024	2018	[83]
EVAtlas	Extracellular Vesicles Atlas	miRNA	Expression	https://guolab.wchscu.cn/EVAtlas/#/ accessed on 5 November 2024	2019	[84]
deepBase	Expression atlas and interactive analysis of ncRNAs from deep-sequencing data	miRNA, circRNA	Expression	https://rna.sysu.edu.cn/deepbase3/index.html accessed on 5 November 2024	2010	[85]
Spliceosome Database	A source of information for the SPLICEOSOME	snRNA	Expression	http://spliceosomedb.ucsc.edu/ accessed on 5 November 2024	2013	[86]
lnCAR	lncRNAs from cancer arrays	lncRNA	Expression	https://www.lncar.renlab.org/ accessed on 5 November 2024	2019	[87]
piRNA-eQTL	PIWI-interacting RNA eQTL Database	piRNA	Expression	http://njmu-edu.cn:3838/piRNA-eQTL/ accessed on 5 November 2024	2021	[88]
miRDB	miRNA target prediction and functional annotations database	miRNA	Gene, genome, and annotation	https://mirdb.org/ accessed on 5 November 2024	2008	[89]
LNCipedia	A comprehensive compendium of human long non-coding RNAs	lncRNA	Gene, genome, and annotation	http://www.lncipedia.org accessed on 5 November 2024	2013	[90]
Unicentral	The non-coding RNA sequences database	miRNA, lncRNA, snoRNA, snRNA	Gene, genome, and annotation	https://rnacentral.org/ accessed on 5 November 2024	2011	[91]
MirGeneDB	The curated microRNA gene database	miRNA	Gene, genome, and annotation	https://master.cloud.mirgenedb.org/ accessed on 5 November 2024	2015	[92]
circRNADb	Circular RNA database	circRNA	Gene, genome, and annotation	http://reprod.njmu.edu.cn/circrnadb accessed on 5 November 2024	2016	[93]
CSCD	Cancer-specific circRNA database	circRNA	Gene, genome, and annotation	http://gb.whu.edu.cn/CSCD/ accessed on 5 November 2024	2018	[94]
piRNAclusterDB	piRNA cluster database	piRNA	Gene, genome, and annotation	https://www.smallrnagroup.uni-mainz.de/piRNAclusterDB/ accessed on 5 November 2024	2012	[95]
snOPY	A small nucleolar RNA orthological gene database	snoRNA	Gene, genome, and annotation	http://snoopy.med.miyazaki-u.ac.jp/ accessed on 5 November 2024	2013	[96]
GeneCaRNA	The human ncRNA database	piRNA, miRNA, lncRNA, snoRNA	Gene, genome, and annotation	https://www.genecards.org/genecarna accessed on 5 November 2024	2021	[97]
piRBase	Database supporting piRNA functional studies	piRNA	Gene, genome, and annotation	http://bigdata.ibp.ac.cn/piRBase/ accessed on 5 November 2024	2022	[98]
miRBase	microRNA database	miRNA	Gene genome and annotation	https://mirbase.org/ accessed on 5 November 2024	2004	[99]
ENCORI/starBase	Encyclopedia of RNA interactomes	miRNA, circRNA, lncRNA	Interaction	https://rnasysu.com/encori/ accessed on 5 November 2024	2011	[100]
DIANA-LncBase	Database for miRNA–lncRNA interactions	miRNA, lncRNA	Interaction	https://diana.e-ce.uth.gr/lncbasev3 accessed on 5 November 2024	2013	[101]
NPInter	An integrated database of ncRNA interactions	miRNA, lncRNA, circRNA, snoRNA, snRNA	Interaction	http://bigdata.ibp.ac.cn/npinter5 accessed on 5 November 2024	2006	[102]
CircNet	Circular RNA regulatory networks in cancers	miRNA, circRNA	Interaction	https://awi.cuhk.edu.cn/~CircNet/php/index.php accessed on 5 November 2024	2016	[103]
miRNet	A visual miRNA-centric network analytics platform	miRNA	Interaction	https://www.mirnet.ca/ accessed on 5 November 2024	2016	[104]

## 6. Clinical Potential of Non-Coding RNAs

### 6.1. Non-Coding RNAs in Cancer Diagnostics

**Figure 3:**
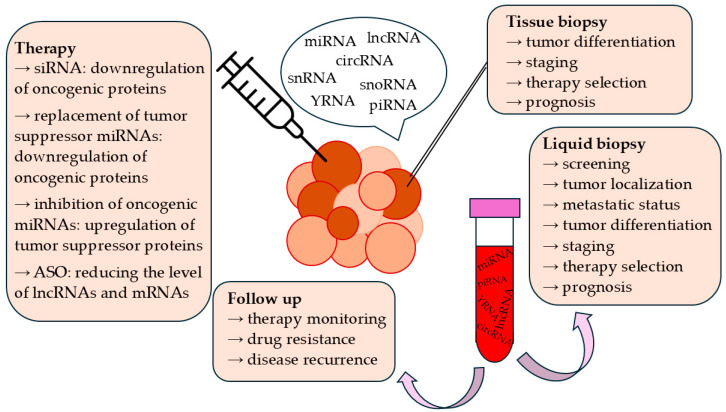
Summary of the clinical potential of ncRNAs. Some ncRNAs are promising biomarker candidates in cancer diagnostics in both tissue and liquid biopsy samples. Their application is also considered to be an effective tool in the personalized therapy of cancer by influencing the expression of proteins involved in cancer progression.

A variety of molecular methods allow ncRNA detection, supporting their use as biomarkers (Figure 3). This is based on observations that the ncRNA signatures of healthy cells differ from those of tumor cells. A large number of ncRNAs have been shown to be associated with various pathophysiological properties of tumors, such as stage, genome instability, metastatic status, or chemotherapeutic resistance. Non-coding RNAs are demonstrating their efficiency as prognostic markers as well. Most reports focus on the diagnostic potential of miRNAs [105,106,107], but lncRNAs [108], circRNAs [109], snoRNAs [110], snRNAs [111], piRNAs [18], YRNAs [112], and vtRNAs [113] are steadily gaining ground. The detectability of cf-ncRNAs in body fluids makes them promising candidates for minimally invasive liquid biopsy [20,23]. Liquid biopsy has several advantages over traditional invasive diagnostic methods: (i) the sampling procedure is simpler, cheaper, and easier to perform; (ii) it is useful in the early detection or screening of a disease; (iii) its repeatability makes it suitable for treatment follow-up, allowing for quick detection of possible drug resistance or recurrence of the disease; (iv) it provides information about the entire tumor, overcoming the issue of genetic heterogeneity, which limits the reliability of tissue biopsies [23,114,115]. Biomarkers can be proteins, circulating tumor cells, or cell-free nucleic acids. The latter group comprises DNA fragments (genomic or mitochondrial) from tumors and ncRNAs discussed above. Cell-free ncRNAs may provide information on the progress of the disease and the localization of the tumor, as well as about the physiology of tumor cells, their sensitivity to chemotherapeutic agents, and the expected prognosis of the disease [20,23]. It is also important to note that ncRNAs have high stability, as they are resistant to degradation by RNases and can survive repeated freeze–thaw cycles. This supports their use as biomarkers in routine diagnostics [20,23].

### 6.2. Non-Coding RNAs in Cancer Therapy

To alleviate or negate the adverse effects of traditional cancer treatment, vivid research is being conducted to develop new drugs and strategies for the targeted elimination of tumor cells while sparing healthy cells [116]. One approach is to increase the expression of tumor suppressor genes or to inactivate oncogenes by ncRNAs (Figure 3). Modifying their expression levels by artificial mimics or inhibitors may meaningfully influence regulatory networks involving a large number of target genes [16,117]. However, there are some limiting factors: (i) degradation by RNases; (ii) inducing the natural immune response through Toll-like receptors (TLR); and (iii) the non-specific “off-target effect”, which means altered translation of an mRNA other than the target mRNA. According to certain studies, some unwanted effects may be reduced by the chemical modification of synthetic ncRNAs [54,106]. For instance, the 2′-OH group of the ribose molecule is an attack point of many nucleases but changing it to a 2′-O-methyl group reduces the extent of miRNA degradation. In addition, replacing phosphodiester bonds with phosphothionate bonds may increase the stability of the molecule and alleviate natural immune responses against it. On another note, the off-target effect caused by the miRNA mimic’s accompanying strand may be reduced if the accompanying strand is composed of two or three smaller strands. This makes the miRNA pieces too short to interact with complementary mRNAs [118,119].

Several different delivery systems are under development or clinical testing. It should be highlighted that miRNAs and lncRNAs are still among the most extensively studied ncRNAs; thus, the bulk of the currently available evidence is related to them [50]. A notable proposed delivery method is the use of adeno-associated viruses. The advantage is that viruses are able to deliver genetic material to the target with high efficiency by infecting mammalian cells; however, certain safety concerns limit their possible clinical application [50,120]. Another approach is the non-viral vector-based RNA delivery method, which includes lipid- and polymer-based systems. Their advantage over viral vectors is that they do not trigger a relevant immune response, but this comes with a lower efficiency of transfection [121]. Exosome-based delivery systems are also known, as some ncRNAs, due to their size, may be artificially introduced into exosomes using standard transfection methods and then delivered to target sites by genetic modification of the surface ligands [50,54]. So far, the most effective delivery systems for miRNA therapy seem to be poly(lactone-glycolic acid) particle (PLGA), natural lipid emulsion (NLE), synthetic polyethyleneimine (PEI), dendrimers, cyclodextrin-based carrier systems, poly(ethylene glycol) (PEG), cytosans, and N-acetyl-D-galactosamine (GalNAc) [117].

## 7. Structure, Biogenesis, and Function of Non-Coding RNAs

While publications on ncRNAs started to increase in the early 2000s, this is now a rapidly developing field with more candidates being identified. According to the most widely used databases, the majority of available publications focus on miRNAs, followed by siRNAs and lncRNAs. These molecules have the potential for therapeutic applications, and there has been active research conducted on the use of miRNAs and/or lncRNAs as biomarkers in routine diagnostics. Fewer studies are available on circRNAs, snRNAs, snoRNAs, piRNAs, vtRNAs, and YRNAs. However, the dysregulation of these molecules in cancer suggests their potential as additional biomarkers for diagnostic applications. Furthermore, understanding the functional details of eRNA- and paRNA-mediated regulation of gene expression might open new avenues in cancer diagnostics in the near future. In this section, we present details on the above-mentioned ncRNAs, including their functions in cancer development and their clinical potential.

### 7.1. MicroRNAs

#### 7.1.1. Biogenesis and Regulatory Functions of MicroRNAs

The biogenesis and regulatory role of miRNAs is well described. As a result of their multistep biogenesis, a mature, single-stranded ~18–23 nt molecule is formed from a double-stranded precursor. MicroRNA coding genes are mainly transcribed by RNA polymerase II or occasionally by RNA polymerase III. Most of the miRNA genes are intergenic and have their own promoters; hence, they are transcribed as independent units, while intronic miRNA genes are usually transcribed together with their host genes and cleaved out of the pre-mRNA by the splicosome. Some miRNAs are organized into clusters and transcribed into one long transcript. During transcription, an approximately 1 kb long hairpin-like structure, the primary miRNA (pri-miRNA), is produced, which consists of the 5′ cap structure and the 3′ poly-A tail and might also contain introns [6,122]. In the nucleus, the Drosha endonuclease cleaves the pri-miRNA molecule to create the ~70 nucleotide-long loop-shaped precursor miRNA (pre-miRNA), which has a single-stranded structure at the 3′ end. The Drosha enzyme complex (microprocessor complex) comprises the ribonuclease III enzyme (Drosha) and the DiGeorge Syndrome Critical Region 8 (DGCR8) RNA binding protein. The pre-miRNA is transported from the nucleus into the cytoplasm by Exportin-5 (Exp5), after which, as a result of a second cleavage mediated by the Dicer enzyme, an approximately 22 nucleotide-long, double-stranded miRNA duplex is produced by removing the terminal loop. Although both strands of the duplex may act as functional miRNAs, only one, the so-called leading strand, is incorporated into the RNA-induced silencing complex (RISC) by binding to the AGO2. The other (“passenger”) strand is broken down. The leading strand is determined by the orientation of the miRNA strand. The strand originating from the 5′-end of the pre-miRNA hairpin is referred to as 5p, while the 3p miRNA originates from the 3′-end. This type of biogenesis is called the canonical pathway [6,122,123]. Some pre-miRNAs dubbed “mirtrons” are excised directly from a shorter intronic region, thus bypassing Drosha-mediated processing [123]. Mature miRNAs integrated into the RISC complex exert their effect by binding to the 3′ UTR of target mRNAs based on the extent of complementarity. High complementarity leads to the degradation of the target mRNA, while lower complementarity leads to inhibition of translation [6,124]. As miRNAs do not require perfect complementarity, as mentioned before, one mRNA molecule may become the target of many miRNAs, and one miRNA may have several different targets. This fact is the basis of the extremely complex regulatory networks enabling post-transcriptional fine-tuning of gene expression [28,125]. Recent studies have revealed that some miRNAs may bind to other regions of mRNAs, including the coding sequence or the 5′ UTR region, which might lead to mRNA degradation, translational repression, or even increased translation, while other miRNAs are thought to interact with non-AGO proteins, enhancing or reducing the efficiency of miRNAs on their mRNA targets. Interestingly, some pri-miRNAs may even encode peptides that have an influence on miRNA expression [6,16].

#### 7.1.2. Role of MicroRNAs in the Regulation of Cancer Progression

**Figure 4:**
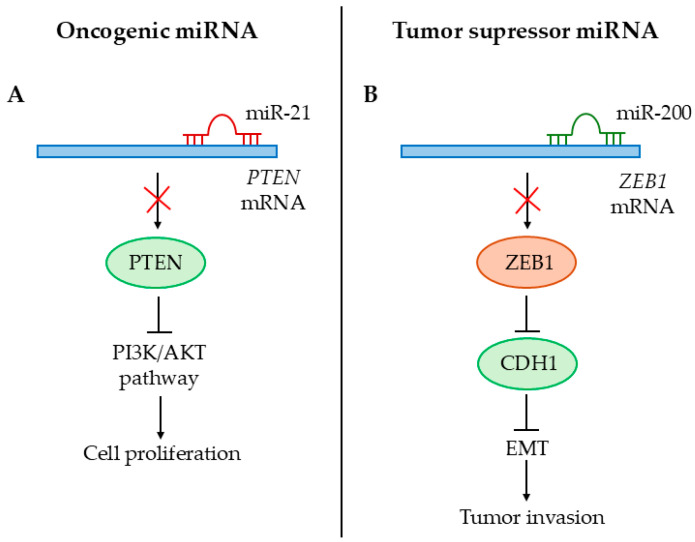
Molecular mechanisms of action of oncogenic and tumor suppressor miRNAs. (**A**) miR-21 has an oncogenic function by targeting *PTEN*, which supports cell proliferation [131]. (**B**) miR-200 family members are known tumor suppressors that inhibit epithelial–mesenchymal transition (EMT)-mediated tumor invasion by targeting *ZEB1/2* [132].

Through their effect on gene expression, miRNAs are involved in the regulation of many biological processes such as cell proliferation, differentiation, migration, apoptosis, cell cycle, and various stress response mechanisms [17]. Abnormal miRNA patterns are observed in many diseases, including cancer. Both intracellular and cell-free forms of miRNAs play a prominent role in the formation and growth of tumors by influencing factors essential for malignant changes. These include the independence from growth signals (e.g., the let-7 family), insensitivity to anti-growth signals (e.g., the miR-17-92 cluster), avoidance of apoptosis (e.g., miR-34a), unlimited replication capacity (e.g., miR-373/373 cluster), promotion of angiogenesis (e.g., miR-210), and tumor invasion and metastasis (e.g., miR-10b) [17]. They may also suppress the immune response directed against tumors by inhibiting the cytotoxicity of T cells or promoting the production of immunosuppressive cytokines [126,127,128]. Based on these findings, miRNAs may act as oncogenes (oncomiRs), tumor suppressors (TS-miR), metastasis-promoters or suppressors (metasta-miR), angiogenesis-promoters (angio-miR), or immunomodulators (immuno-miR) [129,130]. The exact function of several miRNAs has been characterized in various cancer types, including their interacting lncRNA or mRNA partners (Table 2). MiR-21 and miR-200 are two of the most studied miRNAs in cancer. MiR-21 has an oncogenic function that is exerted by targeting *PTEN*. This leads to upregulation of the PI3K/AKT pathway, which supports cell survival and tumor growth (Figure 4A) [131]. MiR-200 family members are well-known tumor suppressors that are involved in the inhibition of epithelial–mesenchymal transition (EMT)-mediated tumor invasion by targeting *ZEB1/2* (Figure 4B) [132].

#### 7.1.3. Possible Clinical Application of MicroRNAs

MicroRNAs are considered promising biomarker candidates in cancer diagnostics, both in tissue samples and liquid biopsies, supported by the observation that miRNA expression patterns are highly different between healthy and cancerous cells [16,23,119]. Due to their cell- and tissue-specific expression, they may provide insight into the origin and type of tumors, the stage of the disease, expected sensitivity to chemotherapy, and, ultimately, prognosis [105,106,107]. They might also be applicable markers for liquid biopsies [20,119,129]. There are several ongoing clinical trials aiming to develop miRNA-based diagnostic tests for various types of cancer [133].

Modifying intracellular expression levels of miRNAs is considered a promising therapeutic approach, typically involving one of two main strategies: (i) miRNA replacement therapy with miRNA mimics with the aim of increasing the level of tumor suppressor miRNAs in the cell or (ii) the use of miRNA inhibitors (antimirs, antagomirs, or sponges) that possess several target sites for miRNAs so they reduce the presence of oncomiRs in the cell [16,119,122]. Another recent approach is to apply small-molecule inhibitors to disrupt the biogenesis of miRNAs [134]. Clinical trials to assess miRNA-based therapeutic strategies are also in progress [134]. MiR-16 is a tumor suppressor showing reduced expression in many tumor types. To replace this molecule, “MesomiR-1” was developed (by EnGeneIC). Its intravenous administration showed promising results in phase 1 clinical trials, and preparations for phase 2 trials are currently underway [135,136]. Some trials (currently undergoing preliminary assessment to enter the clinical stage) focus on strategies against the oncogenic miR-10b [134,135,137]. An miR-155 tumor suppressor antagonist is also in clinical phase 2 clinical trials [16,138]. The application of miR-193a-3p mimic in solid tumors (INT-1B3), miR-16 mimic in non-small lung cancer, miR-34a mimic (MRX34) and anti-miR against miR-155 (Cobomarsen/MRG 106) in various cancer types are also in phase I clinical trials [134]. A modified version of miR-34a with increased stability and activity is also available, and so are delivery strategies allowing ligand targeting and endosomal escape [139,140]. A combination of miRNAs with traditional chemotherapy or radiotherapy may provide additional opportunities for the development of more personalized tumor therapies [119,135].

**Table 2:** The most studied miRNAs in cancer. MicroRNAs might interact with lncRNAs in the regulation of genes involved in cancer progression. In the table, we present examples of experimentally validated miRNA–lncRNA–mRNA interactions.

MiRNA	Interacting lncRNA	Gene	Cancer Type	Reference
let-7a	H19	*CXCR4*, *IL-6*	Cholangiocarcinoma	[141]
let-7b	H19	*CXCR4*, *IL-6*	Cholangiocarcinoma	[141]
miR-1	HOTAIR	*CCND1*	Esophageal Squamous Cell Carcinoma	[142]
miR-7	CCAT1	*HOXB13*, *SPRY4*	Esophageal Squamous Cell Carcinoma	[143]
miR-9	Linc00176	*Myc*	Hepatocellular Carcinoma	[144]
miR-17	circ-ITCH	*p21*	Bladder Cancer	[145]
miR-21	MEG3	*PTEN*	Non-Small Cell Lung Cancer	[146]
miR-22-3p	MALAT1	*AKT*, *CXCR2*	Kidney Carcinoma	[147]
miR-101	CASC2c	*CPEB1*	Astrocytoma	[148]
miR-122	SNHG7	*FOXK2*	Hepatocellular Carcinoma (HCC)	[149]
miR-124	MALAT1	*SIRT1*	Cervical Cancer	[150]
miR-126	HOTAIR	*EGFL7*	Renal Cell Carcinoma	[151]
miR-140-5p	Unigene56159	*Slug*	Hepatocellular Carcinoma	[152]
miR-142	TTN-AS1	*CDK5*	Lung Adenocarcinoma	[153]
miR-143	UCA1	*FOSL2*	Ovarian Cancer	[154]
miR-145	UCA1	*FSCN1*, *ZEB1*, *ZEB2*	Bladder Cancer	[155]
miR-150	ZFAS1	*MMP14*, *MMP16*, *ZEB1*	Hepatocellular Carcinoma	[156]
miR-155	MALAT1	*FBXW7*	Glioma	[157]
miR-195	PVT1	*EZH2*	Cervical Cancer	[158]
miR-200a	TP73-AS1	*HMGB1*, *RAGE*	Hepatocellular Carcinoma	[159]
miR-200b	H19	*MET*	Breast Cancer	[160]
miR-200c	MALAT1	*TGF-beta*	Endometrioid Endometrial Carcinoma	[161]
miR-204	UCA1	*ATF2*	Prostate Cancer	[162]
miR-205	ROR	*ZEB2*	Breast Cancer	[163]
miR-206	RMRP	*FMNL2*, *KRAS*, *SOX9*	Lung Cancer	[164]
miR-210	XIST	*NME1*	Colorectal Cancer	[165]
miR-214	DANCR	*CTNNB1*	Hepatocellular Carcinoma	[166]
miR-221	GAS5	*DKK2*	Breast Cancer	[167]
miR-223	PITPNA-AS1	*PTN*	Lung Squamous Cell Carcinoma	[168]
miR-375	UCA1	*SOX12*	Breast Cancer	[169]

### 7.2. Small Interfering RNAs

#### 7.2.1. Biogenesis and Regulatory Functions of Small Interfering RNAs

Small interfering RNAs are double-stranded ncRNAs with a length of 20–23 nucleotides. Their main mechanism of action is shared with miRNAs, namely, post-transcriptional gene silencing [170,171]. They originate from sh-RNAs or long dsRNAs. The latter case is explained by their sense and antisense strands being transcribed from the same locus of the DNA template, resulting in complementary sequences that eventually form a double-stranded dsRNA. The dsRNAs are then exported through the nuclear pore into the cytosol, where Dicer cleaves them and forms the siRNA duplex consisting of a passenger strand (sense strand) and a leader strand (antisense strand). Then, the siRNA is incorporated into the RISC complex and interacts with the AGO2 component, which results in the unwinding of the duplex and the degradation of the passenger strand. The leading strand, based on complementarity, directs the RISC complex to the target mRNA [170,171]. However, it is important to highlight that the loading efficiency of siRNA into RISC is critical for proper gene silencing. One of the key factors is rooted in the structure of the RNA. The A-form helix fits perfectly and stably, causing degradation of the mRNA molecule, while the B-form helix fits imperfectly and causes RNA interference [171,172]. Although their function is similar to that of miRNAs, a notable difference involves the number of targets—while miRNAs have several, siRNAs only have one [171].

#### 7.2.2. Possible Clinical Application of Small Interfering RNAs

Endogenous siRNAs play a crucial role in silencing transposons and maintaining genome integrity. Some authors have reported differentially expressed siRNAs in tumors [173]. Great effort has been devoted to the development of technologies using siRNAs in cancer therapy. Synthetic siRNAs can be used to specifically silence genes associated with a variety of diseases, including cancer, but they are hindered by low cellular uptake and sensitivity to degradation by nucleases. With chemical modifications, their stability and cell specificity might be improved, and the host’s immune reaction and certain off-target effects might be reduced [170,174]. Many agree that there is great potential in siRNA-based cancer therapy. They include suitability for the targeted silencing of genes that cannot be medicated or are not accessible for small molecules such as antibodies or proteins. They are capable of inhibiting the proliferation of abnormal cells by obstructing angiogenesis and tumor survival, as well as by increasing sensitivity to radio- and chemotherapy [171,172]. Currently, several siRNA-based therapeutic drugs are in phase I or II clinical trials. These include CALAA-01 (Calando Pharmaceuticals), which targets the M2 subunit of ribonucleotide reductase (RRM-2), often overexpressed in solid tumors; Atu027 (Silence Therapeutics GmbH), which targets the N3 protein kinase (PKN3), a critical factor of metastasis in solid tumors; ALN-RSV (Alnylam Pharmaceuticals), which silences the vascular endothelial growth factor (VEGF) and kinesin spindle protein (KSP) genes, leading to complete regression of the tumor in some cases (endometrial cancer); DCR-MYC (Dicerna Pharmaceuticals), which targets the c-MYC oncogene and seems to be suitable for solid tumors and multiple myeloma; siRNA-EphA2-DOPC (M.D. Anderson Cancer Center, Houston, TX, USA), which targets the Ephrin-A receptor 2 (EphA2) gene overexpressed in many advanced cancers; and siG12D-LODER (Silenseed Ltd., Jerusalem, Israel), which targets the KRAS (G12D) oncogene, an important driver in the progression of pancreatic cancer [170,175,176,177,178,179,180].

### 7.3. Long Non-Coding RNAs

#### 7.3.1. Biogenesis and Regulatory Function of Long Non-Coding RNAs

Long non-coding RNA molecules are longer than 200 nt and form a distinct yet diverse group among ncRNAs. Although many lncRNAs have already been identified (according to the GENCODE project, there are approximately 16,000 human lncRNAs, while the NONCODE database contains nearly 100,000 human lncRNA genes), only a few of them have been functionally characterized [7,16]. They may be transcribed in the sense or antisense direction, mainly by RNA polymerase II. Long non-coding RNA genes are found in the introns (intronic lncRNAs), exons of protein-coding genes or pseudogenes (pseudogene-derived lncRNAs), in intergenic regions (lincRNAs), telomeric (telomeric repeat-containing RNAs) and centromeric (centromeric lncRNAs) regions, transcribed ultra-conserved regions (T-UCR), promoters (promoter-associated lncRNAs), enhancers (eRNAs), ribosomal DNA loci (promoter and pre-rRNA antisense (PAPAS)), and 3′ UTR regions (UTR-associated RNAs) [7,16]. Like mRNAs, lncRNAs may contain exons (typically less numerous but longer), and they may also undergo splicing. They often possess a 7-methylguanosine cap at their 5′ ends and a poly-A tail at their 3′ ends but lack a complete open reading frame [7,16,181].

Functionally, they may participate in the genomic, transcriptional, and translational regulation of neighboring and distant genes, influence the maturation and stability of RNA molecules, and modify chromatin structure by directly interacting with DNA, RNA (including mRNAs, circRNAs, and miRNAs), and proteins [7,16,182]. Regulatory mechanisms based on DNA interaction include lncRNA-mediated chromatin regulation, of which three main mechanisms are known: (i) they may interact with chromatin modifiers and recruit them to the promoter region of the target genes, thus activating or repressing their transcription (e.g., HOTTIP); (ii) they may bind chromatin modifiers, preventing them from associating with the promoter region of the target gene (e.g., lncPRESS1); (iii) they may directly interact with chromatin and form three-stranded RNA–DNA hybrids (e.g., R-loops). These are recognized by chromatin modifiers or transcription factors, which in turn activate/inhibit the transcription of the target gene (e.g., TARID) [7,183,184,185]. Another regulation involving interaction with DNA is lncRNA-directed gene silencing, most importantly by XIST as a key regulator of X-chromosome inactivation [186]. Regulatory mechanisms induced by RNA interaction can be observed at several levels. The translation (e.g., by AS-Uchl1) and stability (e.g., by TINCR) of mRNA molecules may be affected by direct binding of lncRNAs, as well as by blocking miRNA-binding sites, thus inhibiting their effect (e.g., by PTB-AS). On another note, lncRNAs may interact with miRNAs and bind them like a sponge (e.g., MALAT1 and PNUTS) [7,16,187]. Furthermore, lncRNAs function as scaffolds by interacting with target proteins (e.g., NRON65 and HOTAIR), and they may also serve as precursors for the production of sncRNAs [16,188].

#### 7.3.2. Role of Long Non-Coding RNAs in the Regulation of Cancer Progression

Similar to miRNAs, lncRNAs may also have oncogenic or tumor suppression functions. Oncogenic lncRNAs promote cell proliferation (e.g., REG1CP—colorectal cancer, SATB2-AS1—osteosarcoma, MALAT1—liver and lung cancer), cell invasion (e.g., PCAT19—prostate cancer, HOTAIR—prostate cancer), and metastasis (e.g., PCAT19—prostate cancer, SATB2-AS1—osteosarcoma, MALAT1), and they may also contribute to evading the immune response (e.g., LINKA) and apoptosis (e.g., XIST—breast cancer, MALAT1—liver cancer), or promote the degradation of tumor suppressors (e.g., LINKA) [189,190,191,192,193,194,195,196,197]. Tumor suppressor lncRNAs may activate the transcription of tumor suppressor genes (e.g., DIRC3—melanoma) by modifying local chromatin structure or reduce the proliferation and invasion of tumor cells (e.g., SATB2-AS1—colorectal cancer, LINC00261—lung cancer, PVT1—breast cancer, MEG3—colorectal cancer). They also participate in DNA damage-induced responses (LINC00261—liver, breast, and gastric cancers) [195,198,199,200,201]. Furthermore, they might increase cancer cell resistance to chemotherapeutic drugs (e.g., NEAT1) [202].

**Figure 5:**
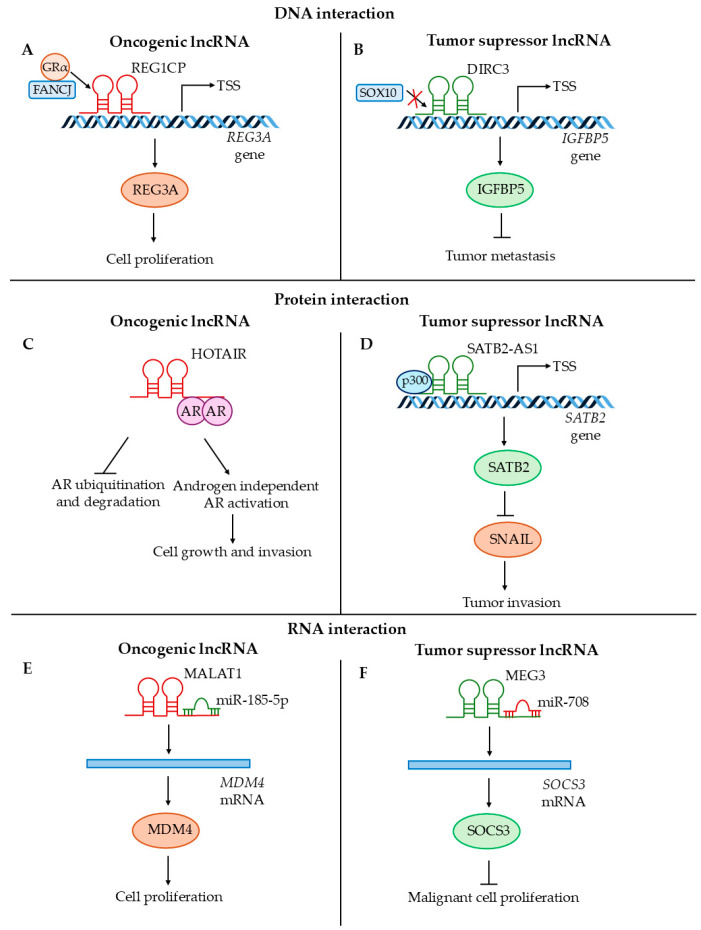
Molecular mechanism of action of oncogenic and tumor suppressor lncRNAs. Long non-coding RNAs can exert their functions via DNA, protein, or RNA interactions. (**A**) The oncogenic REG1CP promotes tumorigenesis by forming an RNA–DNA triplex at the distal promoter region of *REG3A*, thus supporting its glucocorticoid receptor α (GRα)-mediated transcription by tethering FANCJ [194]. (**B**) DIRC3 is a nuclear-tumor-suppressor lncRNA that activates the expression of *IGFBP5* by modulating chromatin structure and preventing SOX10 binding to the regulatory elements of the *DIRC3* locus [198]. (**C**) HOTAIR effects its oncogenic potential by interacting with the androgen receptor (AR) protein, which blocks AR ubiquitination and leads to AR-mediated transcription [190]. (**D**) The tumor suppressor SATB2-AS1 serves as a scaffold for recruiting p300 protein, which promotes *SATB2* transcription [195]. (**E**) MALAT1 exerts its oncogenic function by sponging miR-185-5p, which targets *MDM4* [189]. (**F**) MEG3 acts as a tumor suppressor by sponging miR-708, leading to increased *SOCS3* expression [201]. TSS: transcription start site.

Long non-coding RNAs can exert their oncogenic and tumor suppressor functions by interacting with DNA, proteins, or RNA (e.g., by sponging miRNAs) (Figure 5). REG1CP promotes tumorigenesis by forming an RNA–DNA triplex at the distal promoter region of *REG3A*; this supports its glucocorticoid receptor α (GRα)-mediated transcription by tethering the DNA helicase FANCJ. This leads to increased cell proliferation in colorectal cancer (Figure 5A) [194]. DIRC3 is a nuclear tumor suppressor lncRNA that activates the expression of *IGFBP5* by modulating chromatin structure and preventing SOX10 binding to the regulatory elements of the *DIRC3* locus. This suppresses tumor invasion and metastasis in melanoma (Figure 5B) [198]. Long non-coding RNAs can serve as scaffolds for proteins involved in cancer development that support their survival or improve protein interaction and function. HOTAIR has oncogenic potential via its interaction with the androgen receptor (AR) protein. This interaction blocks AR ubiquitination by MDM2, preventing its degradation. This leads to AR-mediated transcription in the absence of androgen, which supports the growth and invasion of prostate cancer cells (Figure 5C) [190]. SATB2-AS1 has a tumor suppressor function and serves as a scaffold for recruiting p300 protein, which promotes *SATB2* transcription via acetylation of H3K27 and H3K9 at the promoter regions. This results in the blockage of *SNAIL* transcription and inhibition of tumor invasion in colorectal carcinoma (Figure 5D) [195]. Furthermore, lncRNAs often serve as sponges for oncogenic or tumor-suppressor miRNAs. MALAT1 sponges miR-185-5p, leading to increased *MDM4* expression, which supports cell proliferation in non-small cell lung cancer (Figure 5E) [189]. MEG3 acts as a tumor suppressor by sponging miR-708 that targets *SOCS3*. This upregulates SOCS3 expression, which inhibits cancer stem cell growth in colorectal cancer (Figure 5F) [201].

#### 7.3.3. Possible Clinical Application of Long Non-Coding RNAs

A number of promising lncRNAs show altered expression in many tumor types, e.g., PCA3 (in prostate cancer), HOTAIR (breast cancer, laryngeal squamous cell carcinoma, cervical cancer, and urothelial bladder cancer), BCAR4 (colorectal cancer), and MALAT1 (non-small cell lung cancer) [203,204,205,206]. Some lncRNAs have also been associated with metastasis (CCAT2—liver metastasis of colorectal carcinoma; HOTAIR—liver metastasis of gastric cancer and brain metastasis of small cell lung carcinoma) [207,208,209,210].

Long non-coding RNAs also show some promise as therapeutic targets. One strategy is the use of ASOs, which reduce lncRNA levels by binding to them and inducing RNase H-mediated cleavage, leading to premature termination of transcription. To avoid toxicity and degradation by nucleases, they can be chemically modified to enhance their hybridization affinity for target RNAs and reduce any non-specific immunostimulatory activity [7,211]. To date, several mRNA-targeting ASOs have been approved by the FDA and the European Medicines Agency. Examples of these are Mipomersen (Genzyme), Nusinersen (Biogen), Patisiran (Alnylam), Inotersen (Ionis), Eteplirsen (Sarepta), Golodirsen (Sarepta), Givosiran (Alnylam), and Milasen (Boston Children’s Hospital) [212,213,214,215,216,217,218]. Small molecules for targeting lncRNAs or blocking their interaction with proteins or the application of synthetic lncRNA mimics (which serve as decoys for proteins) might also become relevant from a therapeutic perspective in the future [7].

### 7.4. Circular RNAs

#### 7.4.1. Biogenesis and Regulatory Functions of Circular RNAs

Circular RNAs are formed by the back-splicing of linear transcripts (precursor mRNAs or, in some cases, lncRNAs). During this process, the 5′ splice donor site is covalently linked to the 3′ splice acceptor site. They may originate from exons, introns, exon–intron junctions, or intergenic regions of the genome. The majority of circRNAs are assumed to be formed as a result of base pairing between inverted Alu elements upstream and downstream of exons. It is worth noting that their expression is often independent of host genes [8,16,191]. Their function is based on their interaction with miRNAs, mRNAs, and proteins. Circular RNAs may bind miRNAs as miRNA sponges, preventing them from interacting with their target mRNAs. As a result, the circRNA indirectly influences the expression of the target gene (e.g., ciRS-7 contains miR-7 binding sites, while circTDRD3 contains miR-1231 binding sites). Circular RNAs may also bind directly to specific mRNAs, thereby regulating their translation and stability (e.g., the interaction of circZNF609 with CKAP5 mRNA). Interestingly, some circRNAs, such as circCDYL2, are capable of translation [16,219]. Interaction of circRNAs with proteins occurs in four main forms: (i) they may bind them in a spongelike manner in the presence of an RNA-binding protein motif (RBP), thereby regulating their activation, (ii) they may enhance the function of certain proteins (e.g., the interaction between RNA polymerase II and small nuclear ribonucleoprotein U1), (iii) they may also function as protein scaffolds, facilitating the colocalization of enzymes and their substrates, and (iv) they may recruit certain proteins to specific loci (e.g., FECR1 recruits the methylcytosine dioxygenase TET1 to the promoter region of its host gene) [16,219].

#### 7.4.2. Role of Circular RNAs in the Regulation of Cancer Progression

**Figure 6:**
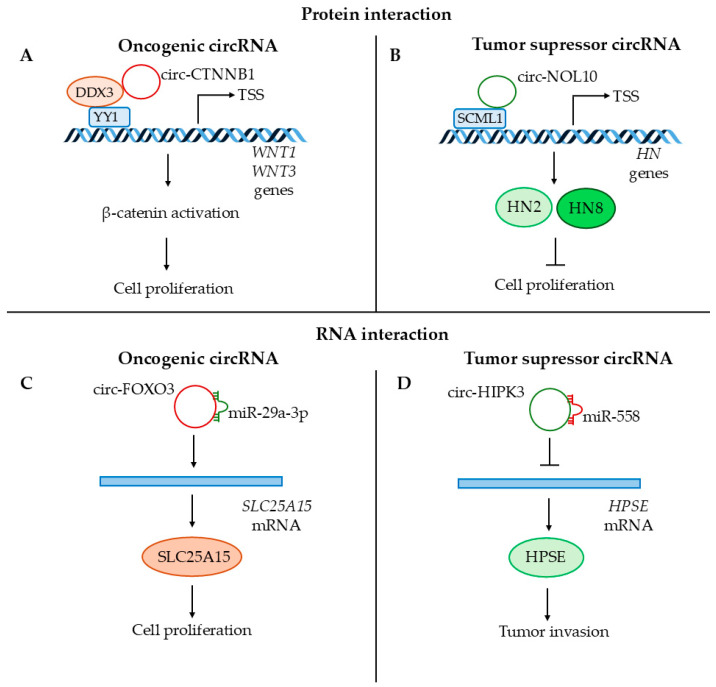
Molecular mechanism of action of oncogenic and tumor suppressor circRNAs exerting their functions via protein and RNA interactions. (**A**) circ-CTNNB1 interacts with DDX3 protein, which facilitates its interaction with YY1 [220]. (**B**) circ-NOL10 inhibits cancer development by promoting the expression of *SCML1* by inhibiting transcription factor ubiquitination. This affects the expression of humanin polypeptide (HN) family proteins [221]. (**C**) circ-FOXO3 exerts its oncogenic function by sponging miR-29a-3p, which leads to upregulation of *SLC25A15* [222]. (**D**) circ-HIPK3 exerts its tumor suppressor function by suppressing heparanase expression by sponging miR-558. This prevents the transport of miR-558 to the nucleus, where it supports the transcription of HPSE [223].

Circular RNAs are also considered to be important in the development of cancer. Interestingly, the same circRNAs may act as both oncogenes and tumor suppressors, depending on the tumor type, stage, and microenvironment of the neoplasm [16]. Circular RNAs might serve as scaffolds for oncogenic or tumor suppressor proteins that support protein interaction or sponge oncogenic/tumor suppressor miRNAs (Figure 6). Circ-CTNNB1 interacts with DDX3 protein, which facilitates its interaction with YY1. This leads to the expression of genes associated with β-catenin activation, which supports cancer progression (Figure 6A) [220], while circ-NOL10 inhibits cancer development by promoting the expression of *SCML1* by inhibiting transcription factor ubiquitination. This affects the expression of humanin polypeptide (HN) family proteins and mitochondrial function in lung cancer (Figure 6B) [221]. Known miRNA sponges include circ-FOXO3, which acts as an oncogene in prostate cancer, and circHIPK3, which functions as a tumor suppressor in bladder cancer. Circ-FOXO3 sponges miR-29a-3p, which targets *SLC25A15*. This leads to the upregulation of *SLC25A15*, which supports cell proliferation (Figure 6C) [222]. Circ-HIPK3 suppresses heparanase expression by sponging miR-558. This prevents the transport of miR-558 to the nucleus, where it supports the transcription of *HPSE*, resulting in decreased tumor invasion (Figure 6D) [223].

#### 7.4.3. Possible Clinical Application of circular RNAs

Various authors have reported circRNAs with altered expression in cancer; moreover, these molecules have also been successfully identified in exosomes [50,192]. For example, circFARSA showed elevated expression in tissue and plasma samples of non-small cell lung cancer patients in one study, while another study demonstrated significantly lower circ-CCDC66, circ-ABCC1, and circ-STIL plasma expression levels in colorectal cancer [224,225]. Other biomarker candidates include SMARCA5 in liver cancer and circ-CDYL in colon, bladder, and triple-negative breast cancers, where circRNA downregulation positively correlates with patient survival. Circ-RNAHIPK3 has been proposed as a promising candidate in glioma as well as prostate, breast, colon, and kidney cancers [192,226].

### 7.5. Small Nuclear RNAs

#### 7.5.1. Biogenesis and Regulatory Functions of Small Nuclear RNAs

Small nuclear RNAs are ncRNA molecules that are around 150 nt in length. They serve as crucial components of spliceosomes and are divided into two large groups. Sm snRNAs (U1, U2, U4, U4atac, U5, U7, U11, U12) are transcribed by RNA polymerase II and associated with seven Sm proteins, while Lsm (Sm-like) snRNAs (U6, U6atac) are transcribed by RNA polymerase III and associated with seven Lsm proteins [4,109]. Polymerase II-driven snRNAs (which are the main spliceosomal snRNAs) have a monomethylguanosine (m7G) cap at their 5′ ends and a 3′ overhang, while polymerase III snRNAs have a post-transcriptional methyl group on the γ-phosphate at their 5′ ends and also have a 3′ overhang. During their biogenesis, these transcripts must go through a maturation process that varies between the two types of snRNAs. While Sm-type pre-snRNAs are exported to the cytoplasm through the nuclear pore complexes with the help of the export factor CRM1, the Lsm-type pre-snRNAs remain in the nucleus [227]. Maturation of Sm-type pre-snRNAs involves the 3′ end getting trimmed by exonucleases and hypermethylation of the m7G-cap. After that, snRNAs connect to Sm proteins through their Sm binding site to form the heptameric Sm ring to stabilize snRNAs; the resulting complex is called Sm-class snRNP. After immature Sm snRNPs are formed, they are reimported into the nucleus, where they initially localize to Cajal bodies and undergo pseudouridylation and 2′-O-methylation by small Cajal body-specific RNAs (scaRNAs) and final assembly steps. After that, the mature spliceosomal snRNPs accumulate in the nuclear speckles. On the other hand, the 2′-O-methylation and pseudouridylation of Lsm snRNAs are assumed to happen in a site-specific manner with the help of snoRNAs. Following this, they form a complex with Lsm proteins to form U4/U6 dimers and then bind the mature U5 snRNP to form pre-assembled U4/U6.U5 tri snRNP for splicing [227,228,229,230].

#### 7.5.2. Role of Small Nuclear RNAs in Cancer Progression

Their dysregulation is associated with the development and progression of tumors due to their ability to increase the level of oncogenes and reduce tumor suppressors. A good example of this is the overexpression of the U1 snRNA, affecting genes involved in the p53 and MAPK signal transmission pathways and in the cell cycle [231]. This is highlighted by the observation that mutated U1 snRNAs may promote the progression of chronic lymphocytic leukemia and cerebellar neuronal cancer through enhancement of CD44, PTCH1, and CCND2 oncogenes, as well as promote the migration and invasion of Hela cells [4,232]. Another example is 7SK snRNA, which contributes to the induction of apoptosis in HEK293T cells when overexpressed [233].

#### 7.5.3. Possible Clinical Application of Small Nuclear RNAs

The use of snRNAs as biomarkers has promising clinical potential. This is supported by reports on the downregulation of U1, U2, and U5 snRNAs in plasma samples from lung cancer patients [109,234]. Furthermore, upregulation of U6 snRNA in cervical carcinoma (tissue samples), elevated levels of RNU2-lf in lung cancer patients (plasma samples), and downregulation of RNU5E-1 in hepatic cell carcinoma cells and tissue samples have also been observed [4,235,236,237].

### 7.6. Small Nucleolar RNAs

#### 7.6.1. Biogenesis and Regulatory Functions of Small Nucleolar RNAs

Small nucleolar RNAs are approximately 60–300 nt in length and constitute a conserved, abundant class of ncRNAs. They occur as monocistronic genes or in polycistronic clusters and are encoded in independent transcription units or found within the introns of protein-coding or non-coding genes. Most snoRNAs (~90%) are embedded in introns (often, those of long, ncRNA genes), but the most abundant snoRNAs, U3 (SNORD3A) and U8 (SNORD118), are transcribed from independent genes [238,239]. Based on their structural motifs and basic structures, they can be divided into two main groups: (i) the C/D box snoRNAs (SNORDs), which are characterized by two highly conserved motifs, the C box (RUGAUGA motif) and the D box (CUGA motif), and (ii) the H/ACA box snoRNAs (SNORAs), which feature two hairpins in their structure connected by the H box (ANANNA), and also contain an ACA box. In addition to these two main groups, a less represented group exists, the so-called scaRNAs, which are associated with Cajal bodies [3,240]. They are transcribed as larger precursors by RNA polymerase II in the proximity of the Cajal body, then undergo various modifications in a protein-associated complex until they reach their mature forms [239]. Independently transcribed human snoRNAs have an m7G cap that must be removed to prevent aberrant snoRNA localization. PARN (for nucleolar localized snoRNAs) and TOE1 (for scaRNAs) nucleases are assumed to be involved in the removal of the 3′ end. Maturation of intron-embedded snoRNAs may occur in a splicing-dependent or -independent way [238,239].

The classic function of snoRNAs is to participate in the post-transcriptional modification of ribosomal and some spliceosomal RNAs. In this context, the C/D box snoRNAs determine the target sites of 2′-O-ribose methylation for methyltransferase fibrillarin to modify specific nucleotides on the target RNA, while H/ACA box snoRNAs direct pseudouridylation by forming a complex (snoRNP) with different groups of nuclear proteins [241,242]. Small nucleolar RNP complexes are mostly known for influencing rRNA folding and stability by interacting with target RNAs through complementary base-pairing during pre-rRNA synthesis in the nucleolus. Other canonical functions of these ncRNAs include stepwise nucleolytic processing of the rRNA transcript and guiding the post-transcriptional modification of snRNAs. It is also thought that they may influence mRNA stability as post-transcriptional regulators [241,242].

#### 7.6.2. Role of Small Nucleolar RNAs in Cancer Progression

More and more reports are pointing to their role in cell regulation and in the development, progression, and metastasis of cancer. Some results suggest that certain snoRNAs may act just like miRNAs [3,243]. Examples of the latter are the ACA42 and ACA45 sncRNAs, which may downregulate CDC2L6 in a manner similar to miRNAs [3,244]. They may play pivotal roles in carcinogenesis, exerting both oncogenic and tumor suppressor functions through the regulation of oncogenic signaling pathways. Affected processes include tumor cell proliferation (e.g., SNORA71A and SNORD78—lung cancer; SNORA71B—breast cancer; SNORD89—ovarian cancer), migration (e.g., SNORA7A—lung cancer; SNORD1C—colorectal cancer; SNORA42—hepatocellular carcinoma), and invasion (e.g., SNORA47—lung cancer; SNORD105B—gastric cancer), as well as the EMT (e.g., SNORD78—lung cancer; SNORA71A—breast cancer). In addition, they may participate in the inhibition of apoptosis (e.g., SNORA80E—lung cancer) and may be associated with poor prognosis (e.g., SNORA7B—breast cancer; SNORD52—hepatocellular carcinoma) [245,246,247,248,249,250,251,252,253,254,255,256,257]. Furthermore, a study showed that SNORD33, SNORD66, and SNORD76 have higher expression levels in plasma samples of non-small cell lung cancer patients [3,258]. These results suggest that these molecules may have potential use as biomarkers in cancer diagnostics.

#### 7.6.3. Possible Clinical Application of Small Nucleolar RNAs

The role of snoRNAs in carcinogenesis also suggests that they could be applicable in cancer therapy, for which several strategic approaches have been developed, including the application of shRNAs or their silencing by ASOs or CRISPR/Cas9. An example of inhibition by ASOs is the repression of the U3 and U8 snoRNAs, suppressing tumorigenicity in lung (H1944) and breast (MCF-7) cancer cells [242,259]. The use of shRNA was investigated in hepatocellular carcinoma cells (SK-HEP-1 and HCCLM3), where snoU2_19-shRNAs effectively inhibited cell proliferation and tumor progression [242,260]. Removal of SNORA50A and SNORA50B snoRNAs by CRISPR/Cas9 technology resulted in increased tumorigenicity in melanoma (CHL-1) and lung (A549; NCI-H23) cancer cells [242,261]. Another approach might be the use of a so-called snoRNA gene expression regulator (snoRNA modulator of gene expression, snoMEN), which refers to a snoRNA-derived vector that may prove to be beneficial in targeted cancer therapy [242]. However, we should also highlight that the applicability of snoRNAs in cancer therapy is still a largely uncharted area.

### 7.7. Enhancer RNAs

#### 7.7.1. Biogenesis and Regulatory Functions of Enhancer RNAs

Enhancer RNAs are ~0.5–2 kb ncRNAs transcribed by RNA polymerase II from active enhancers. Their transcription levels are thought to be highly dependent on the binding strength of this protein. Their transcription also requires specific coactivators and transcription factors, as well as the formation of a suitable chromatin loop [11,262]. Due to the limited nature of available evidence, understanding their biological functions is still largely confined to the field of hypothesis, and we can only make speculative assumptions about their future applicability in the clinic. Their main function is thought to be connected to the regulation of gene expression, which may be exerted (i) through enhancement of transcription by trapping transcription factors (e.g., YY1), (ii) by contributing to the formation of chromatin loops through their interaction with the mediator and cohesion complexes, (iii) by playing a more direct role in transcription by stimulating RNA polymerase II pause release through multivalent interactions with the negative elongation factor (NELF). On the other hand, they may also serve as markers of enhancers [11,19,263].

#### 7.7.2. Role of Enhancer RNAs in Cancer Progression

Some authors have observed a tissue-specific expression of eRNAs and their role in several diseases, including cancer [19,262]. Some eRNAs are thought to be involved in cancer development and progression; for example, the estrogen-associated SMAD7e may contribute to carcinogenesis in the bladder, and reduction of its expression may initiate apoptosis and reduce tumor cell invasion [264], while TBX5-AS1 may affect the progression of non-small cell lung cancer through the regulation of the PI3K/AKT pathway [262,265]. CCAT1, and specifically its CCAT1-L isoform, is able to upregulate the MYC proto-oncogene in many tumor types (e.g., colorectal cancer) by interacting with CTCF and modulating chromatin conformation [266]. Furthermore, a study showed that some m6A-modified eRNAs (e.g., MLXIPe) may promote resistance to radiotherapy in bone-metastatic prostate cancer both in vitro and in vivo [267].

#### 7.7.3. Possible Clinical Application of Enhancer RNAs

Many eRNAs show a strong cancer-type-specific expression pattern, which suggests the applicability of these molecules as diagnostic and prognostic markers. For example, TAOK1e (renal clear cell carcinoma), EN1e (breast cancer), CELF2e (stomach adenocarcinoma), APH1Ae (liver hepatocellular carcinoma), and SPRY4-AS1 (hepatocellular carcinoma, glioblastoma multiforme, adrenocortical carcinoma, brain lower grade glioma, and mesothelioma) may show promise as marker candidates, depending on the results of future studies [268,269].

### 7.8. Promoter-Associated RNAs

#### 7.8.1. Biogenesis and Regulatory Functions of Promoter-Associated RNAs

Promoter-associated RNAs are ncRNA molecules that are transcribed in the sense or antisense directions from a region within a few hundred bases of the transcription start site. On the basis of their size, they may be classified as promoter-associated long RNAs (PALRs), which are longer than 200 nt, or promoter-associated small RNAs (PASRs), which are shorter than 200 nt [10,270,271]. Functionally, they may regulate the transcription of neighboring genes as cis-acting elements through various activating or inhibitory mechanisms. These mechanisms include acting as a scaffold for proteins involved in chromatin remodeling or transcription, participating in the regulation of CpG island methylation, or influencing chromatin structure and recruiting transcriptional regulators [10,270].

#### 7.8.2. Role of Promoter-Associated RNAs in Cancer Progression and Possible Clinical Application

As with other ncRNAs, paRNAs may be involved in various carcinogenesis processes. PaRNA_CCDN1 may influence the rate of cell proliferation in cervical cancer via inhibition of cyclin D1 expression [10,272]. In the case of gastric cancer, paRNA_Ets-1 promotes the activation of ERG-related transcriptional processes by affecting the physical interaction between the NONO RNA-binding protein and the ERG transcription factor [273]. The same paRNA may contribute to the growth, invasion, and metastasis of tumor cells in neuroblastoma [10,274]. Other examples include FOXCUT, which shows a positive correlation with the expression of Forkhead box C1 (FOXC1), a transcription factor implicated in a large number of cancer types (e.g., esophageal squamous cancer, oral squamous cell carcinoma, nasopharyngeal carcinoma, and basal-like breast cancer) [275,276,277,278]. Other examples are HIF2PUT, which positively correlates with expression of the hypoxia-inducible factor-2α (HIF-2α) in osteosarcoma; paRNA_CDH1, which may promote the development of prostate cancer through the transcriptional inhibition of E-cadherin (CDH1); paRNA_VIM, which enhances vimentin transcription by achieving an open chromatin state through the formation of the R-loop, as observed in colon and breast cancers; and Khps1, which may promote the expression of the E2F1-mediated SPHK1 proto-oncogene in cervical cancer, liver cancer, and osteosarcoma [279,280,281,282]. In addition, these ncRNAs seem to show a degree of tissue specificity, and their differential expression has been confirmed in various tumor lesions, hinting at their potential usefulness as biomarkers in the clinic [10].

### 7.9. YRNAs

#### 7.9.1. Biogenesis and Regulatory Functions of YRNAs

In the human genome there are four highly conserved YRNA genes, which are located on the 7q36 chromosome and clustered at a single locus. The products of these genes are the 112 nt YRNA1 (RNY1), the 101 nt YRNA3 (RNY3), the 93 nt YRNA4 (RNY4), and the 83 nt YRNA5 (RNY5), with a stem-loop structure. In addition, YRNA-derived fragments (YsRNAs) are also known to be formed by caspase-3-dependent degradation of YRNAs in apoptotic cells as a result of UV exposure with the help of the RNAse L enzyme and also by poly I:C-mediated activation of the innate immune system [23,112,283]. The biogenesis of YRNAs is still poorly understood overall, but some details are clear. They are transcribed by RNA polymerase III, and transcripts are exported into the cytoplasm by exportin-5 and Ran GTPase [112,283]. It is also known that YRNAs may bind to La- and Ro60 proteins during their biogenesis, which may increase their stability and resistance to nucleases, as well as promote their nuclear export. The binding of these proteins to YRNAs takes place at special sites on the stem-loop structure, as YRNA molecules consist of highly conserved upper and lower stems, a less conserved loop domain that carries protein-binding sites (e.g., nucleolin, ZBP1, and PTB-polypyrimidine tract-binding protein), and a polyuridine tail, which all have different functions [283,284,285]. The lower stem domain contains the Ro60 binding site, which is essential for the formation of Ro60–YRNA complexes [283]. These complexes not only participate in the transport of YRNA into the cytoplasm, but also in the regulation of cellular stress responses, the initiation of DNA replication, and RNA quality control. The loop domain modulates chromatin association, and the proteins associated with the binding sites in this region may modify the intracellular localization of Ro60 proteins and their special cellular functions. Our current understanding of the details is limited by the lack of reported evidence. The upper stem domain is responsible for the initiation of DNA replication and the formation of new DNA replication forks, while the polyuridine tail contains La-protein binding sites responsible for the nuclear retention of YRNA, and together with the Ro60 protein, they protect YRNAs against degradation by exonucleases [112,283].

#### 7.9.2. Role of YRNAs in Cancer Progression and Possible Clinical Application

Similar to other ncRNAs, YRNAs and YsRNAs also play relevant roles in carcinogenesis, as suggested by observations that the presence of RNY1 and RNY3 may contribute to increased cell proliferation and that RNY5 might be capable of influencing the microenvironment of tumors, thereby promoting tumor progression, while on the contrary, it may also induce apoptotic pathways [286]. Their altered expression has already been described in several tumor types, in tissue, FFPE, and blood samples alike. Among others, elevated expression of the following has been detected: RNY3 and RNY4 in clear cell renal cell carcinoma (tissue and blood serum); RNY1 and RNY3 in pancreatic ductal adenocarcinoma (FFPE) and in colon cancer; RNY1, RNY3 (cell culture), and RNY4 (blood serum) in colon cancer; and RNY1 (FFPE) in cervical cancer. Downregulation of the following has also been identified: RNY1, RNY3, RNY4, and RNY5 in prostate (tissue) and bladder cancer (tissue) and RNY1 (blood serum) in head and neck squamous cell carcinoma. Elevated expression of Ys4RNA has been demonstrated in lung cancer. Altered expression of some YsYRNAs has also been demonstrated in gliomas, breast cancer, and neck squamous cell carcinoma [23,112,284,286,287,288,289,290,291,292,293,294]. Based on these studies, YRNAs are another RNA species showing some promise as biomarker candidates, depending on the results of future testing.

### 7.10. Vault RNAs

VtRNAs are 88–140 nt long ncRNAs that originate from the VTRNA-1 (vault RNA1-1, vault RNA1-2, and vault RNA1-3) and VTRNA-2 (vault RNA2-1) loci found at chromosome location 5q31 and are transcribed by RNA polymerase III [295,296]. Although available evidence on vtRNAs’ functions is quite limited, it is clear that they are involved in apoptosis, autophagy, proliferation, cell differentiation, mRNA regulation, DNA repair, nuclear transport, drug resistance, intracellular detoxification, cell–cell communication, and in certain immune reactions based on their association with ribonucleoproteins, or through RNA–ligand, RNA–RNA, or RNA–protein interactions. Furthermore, they also presumably serve as miRNA precursors [295,297,298]. The majority of studies available in the literature focus on their roles in viral infections and cancer. It was observed that the overexpression of certain vtRNAs parallels increased resistance to mitoxantrone in glioblastoma, leukemia, and osteosarcoma [299]. Another study suggested that VTRNA1-1 may enhance tumorigenesis and chemoresistance via lysosomes in hepatocellular carcinoma [296].

### 7.11. PIWI-Interacting RNAs

#### 7.11.1. Biogenesis and Regulatory Functions of PIWI-Interacting RNAs

PIWI-interacting RNAs are ncRNAs ranging between 21 and 34 nt in length. They originate from single-stranded precursors and, unlike miRNAs, are produced via a Dicer-independent mechanism. They are transcribed by RNA polymerase II from the so-called piRNA clusters (single- or double-stranded), which mostly include intergenic loci containing repetitive elements. However, a few of them originate from the 3′ UTR of mRNAs or lncRNAs. A noteworthy difference between single- and double-stranded piRNA clusters is that the former have their own promoters while the latter are transcribed from both strands using the promoters of nearby coding sequences [9,300]. The biogenesis of piRNAs involves two interconnected pathways: primary (synthesis) and secondary biogenesis, also known as the ping-pong cycle (amplification). During the maturation of piRNAs, transcripts are first exported from the nucleus into the cytoplasm. During the primary pathway, precursor piRNAs are cleaved with the help of the Zucchini (Zuc) riboendonuclease and its cofactors, during which ca. 25 nt long pre-piRNAs are created with uridine at their 5′ ends. Pre-piRNAs are then incorporated into PIWI (P-element-induced wimpy testis) proteins. The piRNA/PIWI complex may migrate back to the nucleus and bind to target transcripts or form complexes with the AGO3 and Aub (Aubergine) proteins. Through Zuc-mediated cleavage and complementary targeting between AGO3 and Aub, new piRNAs are continuously generated in an amplified manner during the ping-pong cycle, which may increase the abundance and diversity of piRNAs [9,301,302].

The primary role of piRNAs is to maintain the integrity of the genome through silencing of transposons, but they are also known to participate in epigenetic regulation (e.g., via histone modification or DNA methylation) as well as regulation of gene expression, genome rearrangement, spermatogenesis, and anti-viral responses [9,302]. Silencing of transposons and gene expression regulation may occur (i) directly by binding piRNA/PIWI complexes to the transposons, (ii) post-transcriptionally by cleaving the mRNAs or regulating their stability, (iii) through epigenetic mechanisms, e.g., by recruiting methylation and acetylation factors, (iv) at the translational level, and (v) post-translationally by directly binding to the encoded protein and influencing its stability [9,302,303]. It has also been shown that viral sequences may be incorporated into piRNA clusters; thus, some piRNAs are capable of repressing viral gene expression and promoting responses against invading viruses [9].

#### 7.11.2. Role of PIWI-Interacting RNAs in the Regulation of Cancer Progression and Possible Clinical Application

A number of studies have confirmed the association between piRNAs and carcinogenesis through various mechanisms. Like other ncRNAs, they may have both oncogenic and tumor suppressor functions, affecting proliferation (e.g., piR-55490—lung cancer; piR-823—gastric cancer; piR-52207—ovarian cancer), differentiation (e.g., piR-1245—colorectal cancer), progression (piR-54265—colorectal cancer), apoptosis (e.g., piR-651—lung cancer; piR-33733—ovarian cancer), and metastasis (piR-32051—kidney cancer; piR-54265—colorectal cancer) of cancer cells [304,305,306,307,308,309,310]. PIWI-interacting RNAs are detectable in body fluids (primarily in plasma and serum), and their altered expression is often seen in cancer patients (both in tissue and blood samples); thus, they might serve as biomarker candidates. Several cell-free piRNAs have been reviewed previously [311] and implicated in malignant diseases: in colorectal cancer (e.g., piR-5937; piR-54265), breast cancer (e.g., piR-651, piR-17458), gastric cancer (e.g., piR-019308, piR-004918), lung cancer (e.g., piR-hsa-164586, piR-hsa-5444), and cholangiocarcinoma (e.g., piR-14090389) [311,312,313,314,315,316,317,318,319]. Additionally, earlier studies showed that piR-34736, piR-35407, piR-36249, piR-34377, and piR-36318 are typically downregulated, while piR-651, piR-36026, piR-20582, piR-932, piR-36743, piR-021285, and piR-31106 are upregulated in breast cancer [302]. Their different expressions might support their cancer diagnostic potential.

## 8. Conclusions and Future Perspectives

While ncRNAs are a hot topic in current research, especially in cancer, the field has only been partially explored. Available evidence supports the potential of these molecules (especially that of miRNAs and lncRNAs) as biomarkers or therapeutic agents in the clinic. However, the implication of RNA-based cancer biomarkers in routine diagnostics still faces many challenges. On the one hand, available detection methods are still not sensitive enough to provide reliable results on ncRNA expression—especially in the case of cf-ncRNAs—which makes standardization difficult for routine diagnostics. On the other hand, ncRNA expression is affected by several physiological factors that result in their high variability among patients. The identification of biomarker candidates was also hampered by the fact that early studies mainly focused on clinical samples that were further biased by tumor heterogeneity and by the limited availability of healthy tissues for use as controls. These studies often led to contradictory results—the same miRNA was concluded as a tumor suppressor in one study and an oncomiR in another in the same type of cancer. Furthermore, several ncRNAs considered essential in cellular physiology show altered expression under various conditions that limit their application as disease-specific biomarkers. These obstacles might be overcome by understanding the exact regulatory role of single ncRNAs using in vitro and in vivo studies. It is also becoming increasingly clear that studying standalone ncRNAs is not enough. Non-coding RNAs operate in complex and intricate networks, suggesting that multivariate diagnostic tests analyzing various ncRNAs together with mRNA and protein targets might have better diagnostic reliability. This may open the way to a better understanding of ncRNA-mediated regulatory networks in signaling pathways involved in cancer progression, which might support the development of new strategies in cancer therapy. While the future remains unpredictable, the authors of this review argue that ncRNA biomarkers are poised to gain a foothold in the near future as supportive tools in clinical diagnostics and follow-up. However, true breakthroughs will likely only come with the rise of system biology, where genomic and expression data are integrated to obtain a complete and personalized picture for each patient.
